# Methods of coping and burden of illness: Cause or confound?

**DOI:** 10.4103/0019-5545.31523

**Published:** 2007

**Authors:** Chittaranjan Andrade

**Affiliations:** National Institute of Mental Health and Neurosciences, Bangalore - 560 029, India. E-mail: andrade@nimhans.kar.nic.in

Sir,

I read with much interest the article of Creado *et al*[1] which demonstrated, among other findings, that there were significant correlations between methods of coping and burden of illness among the caregivers of patients with chronic schizophrenia. Specifically, higher escape avoidance, fatalism and passivity scores were associated with higher burden of illness, and higher expressive action and problem-solving scores were associated with lower burden of illness. Creado *et al*[1] concluded that expressive action and problem-solving are methods of coping which can decrease burden of illness, whereas escape avoidance, fatalism and passivity are methods of coping which can increase burden of illness.

This conclusion, though intuitively appealing, is not necessarily correct. An equally credible argument is that patients who are more severely ill increase the caregiver burden of illness and provoke maladaptative methods of coping, such as escape avoidance, fatalism and passivity, whereas patients who are less severely ill decrease the burden of illness and allow the unfolding of more adaptative methods of coping, such as expressive action and problem-solving.

Causality can never be proven from statistical associations. If variables A and B are significantly correlated, A may cause B, B may cause A or A and B may both be influenced by a third, measured or unmeasured, variable. In the last-mentioned instance, the third variable is known as a confounding variable. Creado *et al*[1] opted to interpret their data on the basis of direct causality. They did not consider that severity of illness could act as a confound.

Several criteria have been outlined to help researchers interpret causality. These include the temporal sequence of the variables, the strength, consistency and specificity of the association, the presence of a dose-response type of relationship, and the existence of a credible explanation. However, the most definitive method of determining causality would stem from research that is based on randomized, controlled designs.[2]

So, do the findings of Creado *et al*[1] reflect cause or confound? Although the analyses which the authors present in Table 6 in their article suggest that a confound is more likely, the univariate tests and the low statistical power of the analyses render the results inconclusive. So, there is no way of being certain, especially as it is possible that both cause and confound may be involved. At least two methods of analysis could improve an understanding of the data:

1. A backward, stepwise, multiple regression analysis which examines the effects of measures of illness severity and measures of coping on burden of illness.[3] This analysis would provide an estimate of the magnitude and significance of the unique contribution of each independent variable to the burden of illness.
2. A path analysis which examines chains of influence among variables, and whether the data are consistent with the specified model of causality.[4] Several paths towards increased burden can be tested, including one in which burden of illness is independently influenced by illness severity and coping strategies, and one in which illness severity influences coping, which in turn influences burden. In path analysis, the interdependence among independent variables can also be studied.

It is hoped that these digressions on statistical and research methods guide these and future authors in their approach to hypothesis-setting and data analysis.
